# Valorization of Pumpkin Byproducts: Antioxidant Activity and Carotenoid Characterization of Extracts from Peel and Filaments

**DOI:** 10.3390/foods12214035

**Published:** 2023-11-05

**Authors:** Nicola Pinna, Federica Ianni, Roberto Selvaggini, Stefania Urbani, Michela Codini, Luca Grispoldi, Beniamino Terzo Cenci-Goga, Lina Cossignani, Francesca Blasi

**Affiliations:** 1 Department of Pharmaceutical Sciences, University of Perugia, 06126 Perugia, Italy; nicola.pinna@studenti.unipg.it (N.P.); federica.ianni@unipg.it (F.I.); michela.codini@unipg.it (M.C.); francesca.blasi@unipg.it (F.B.); 2 Department of Agricultural, Food and Environmental Sciences, University of Perugia, 06126 Perugia, Italy; roberto.selvaggini@unipg.it (R.S.);; 3 Department of Veterinary Medicine, University of Perugia, 06126 Perugia, Italy; luca.grispoldi@unipg.it (L.G.); beniamino.cencigoga@unipg.it (B.T.C.-G.)

**Keywords:** crop, bioactives, extraction, HPLC-DAD analysis, antioxidant activity, functional foods, waste

## Abstract

Pumpkin (*Cucurbita* sp.) represents an unquestionable source of valuable nutrients and bioactive compounds having a broad spectrum of health-promoting effects. The goal of this work was to characterize the byproducts (peels and filaments) of different pumpkin varieties belonging to *C. moschata* (Butternut, Lunga di Napoli, Moscata di Provenza, and Violina rugosa) and *C. maxima* (Delica, Delica vanity, Hokkaido, and Mantovana) species in terms of total carotenoid content, antioxidant activity, and carotenoid profiling. The research revealed that peels and filaments were a good source of β-carotene and other non-esterified carotenoids, as well as esterified carotenoids. Considering the growing market demand for safe and healthy food products, pumpkin byproducts, having also an interesting antioxidant bioactivity, could be useful in the development of novel functional products.

## 1. Introduction

Pumpkin, belonging to the Cucurbitaceae family, is one of the most common fruits in the world, grown in Western countries, and its consumption is associated with nutritional benefits as well as health properties [1]. Pumpkin has been identified as an ancient crop, easily adapted to particular soil and atmospheric circumstances. Hosen et al. [2], reviewing on *Cucurbita* spp., declared the pumpkin as a crop to mitigate food and nutritional challenges [2]. Pumpkin processing for industrial applications is responsible for the massive accumulation of non-edible parts (i.e., peels, seeds, and filaments). All of this waste represents a cheap source for the development of new functional foods and nutraceuticals, while helping to minimize waste and environmental effects [3].

Currently, filaments and peels are discarded when pumpkins are processed for oil production and utilized for cooking. These byproducts could be valorized for their richness in bioactive compounds, in particular fiber, phenols, and carotenoids, showing potential health benefits. Recently, Hussain et al. [4] studied the peel, flesh, and seeds of *C. maxima* pumpkins highlighting the presence of various nutritional constituents involved in numerous health properties. Interestingly, Salami et al. [5] carried out an extraction of carotenoids from pumpkin peel using supercritical CO_2_ and subcritical water technology. An evaluation of phytochemical, antioxidant, and burn-wound-healing activities of *C. moschata* peel was carried out by Bahramsoltani et al. [6], while Leichtweis et al. [7] explored the phenolic compound composition and antimicrobial/antioxidant properties of pumpkin peel. Based on this literature, it must be put into evidence that pumpkin filaments are waste that is still poorly studied.

All pumpkin parts represent an interesting source of bioactive compounds for humans, including carotenoids. Numerous epidemiological studies suggest that a suitable carotenoid dietary intake reduces the risk of the major diet-related chronic diseases, including cardiovascular, neurological, and cancer diseases [8]. Nowadays, innovative extraction methods, such as ultrasound- (UAE) and microwave- (MAE) assisted extraction techniques, are of increasing interest for isolating new bioactive compounds, including carotenoids, from foods and agri-food waste [9,10]. Therefore, taking into account the context of a circular economy, peels and filaments are also interesting sources of carotenoids to recover using innovative green extraction technologies [4,11].

In a recent paper, UAE was defined as the most performing unconventional extraction method used to isolate carotenoids from pumpkin pulp by using a binary mixture [12]. Based on this result, pumpkins of eight varieties were studied for their content of carotenoids, antioxidant activity, and carotenoid composition. The profiling of isolated compounds was analyzed using high-performance liquid chromatography coupled with a diode array detector (HPLC-DAD). To the best of our knowledge, this is the first paper presenting quantitative data on the food carotenoid composition of the peel and filaments of eight different pumpkin varieties. Generally, the quantification is mostly concentrated on pro-vitamin A carotenoids, and it is limited to pumpkin pulp [13].

## 2. Materials and Methods

### 2.1. Plant Materials

Pumpkins of eight different varieties, four from *C. moschata* (Butternut, Lunga di Napoli, Moscata di Provenza, and Violina rugosa) and four from *C. maxima* (Delica, Delica vanity, Hokkaido, and Mantovana) species, were collected in October 2021 in the Umbria region (Italy). The peel was removed using a sharp knife, while filaments and seeds were manually separated. Then, the peel and filaments were chopped into small pieces and placed into separate stainless-steel containers. Then, they were dried in a conventional ventilated oven (Binder, Series ED, Tuttlingen, Germany) until constant weight at 40 °C. The obtained parts (dried peels and dried filaments) were ground using a grinder. A fine powder was obtained and stored at room temperature in amber glass vials, protected from light and humidity. The obtained powders were subjected to color analysis, while an aliquot was used for the extraction and the following analyses.

### 2.2. Reagents

2-methylpropionamidine)dihydrochloride (AAPH), 2,2′-azino-bis(3-ethylbenzothiazoline-6-sulphonic acid) diammonium salt (ABTS), (±)-6-hydroxy-2,5,7,8-tetramethylchromane-2-carboxylic acid (Trolox), and fluorescein sodium salt were obtained from Sigma-Aldrich (Milan, Italy). Ultrapure acetonitrile (ACN), formic acid (FA), isopropanol (IPA), water (H_2_O), methanol (MeOH), and methyl tert-butyl ether (MTBE) of HPLC and UHPLC (ultra-high-performance liquid chromatography)-MS grade were acquired from Carlo Erba Reagents (Milan, Italy). The other solvents (hexane, IPA) were purchased from VWR (Milan, Italy).

Lutein (≥92%) was obtained from Extrasynthese (Genay, France). β-carotene (>97.0%) was purchased from TCI (Tokyo Chemical Industry) chemicals (Toshima, Tokyo, Japan). Zeaxanthin dipalmitate, used as a standard, was isolated from a hydroalcoholic extract of goji berries using HPLC [14]. On this basis, a small-scale isolation process via consecutive injections of the goji extract was performed. A sufficient amount (1.5 mg) of zeaxanthin dipalmitate, in pure form, was obtained. It was used to build the calibration curve and to carry out the quantification of esterified (mono- and di-) carotenoids.

### 2.3. Moisture Content

The moisture content was determined according to the method n. 925.10 of the Association of Official Analytical Chemists (AOAC) [15].

### 2.4. Color of Pumpkin Peels and Filaments

The color of pumpkin peel and filament powders was evaluated using the CIELAB parameters (L*, a*, b*) as described in a previous manuscript [16]. The chroma (C*) and hue angle (H*) LCH parameters were calculated as follows:

Chroma (C*) = (a*^2^ + b*^2^)^1/2^

Hue (H*) = arctan (b*/a*)

where a* is the red/green coordinate, and b* is the yellow/blue coordinate.

### 2.5. Extraction of Carotenoids and Determination of Total Carotenoid Content (TCC) of Pumpkin Peel and Filaments

The procedures for carotenoid extraction from the peel and filaments as well as the spectrophotometric determination of the TCC value of carotenoid extracts were carried out following the conditions optimized in a previous paper [12].

The carotenoids were extracted for 30 min at 45 °C by using a sonication bath (mod. AU-65, ArgoLab, Carpi, Italy) and hexane:isopropanol (60:40, *v*/*v*) as a solvent.

The TCC was determined by using a spectrophotometer (Lambda spectrophotometer, PerkinElmer, Inc., Waltham, MA, USA) set at 450 nm and dilutions (0.001–0.005 mg/mL) of the β-carotene standard. The TCC was expressed as μg β-carotene equivalents per gram of dry weight (μg β-CE/g DW) [12].

### 2.6. In Vitro Antioxidant Activities

#### 2.6.1. Free-Radical-Scavenging Activity Using ABTS (ABTS Assay)

The ABTS procedure for the determination of the antioxidant properties of carotenoid extracts was carried out using a method developed in a previous work [12]. The antioxidant capacity was expressed as μg Trolox equivalents per gram of dry weight (μg TE/g DW).

#### 2.6.2. Oxygen Radical Absorbance Capacity (ORAC) Assay (ORAC Assay)

The ORAC assay was carried out according to a procedure described by Persichetti et al. [17]. The measure of the fluorescence decay was carried out using a high-performance plate reader (FLUOstar Optima, BMG LABTECH GMbH, Offenburg, Germany) on the spectrofluorometer at excitation and emission wavelengths of 485 and 520 nm, respectively. The final ORAC values, reported as the equivalent concentration of Trolox (µg TE/g) that produces the same level of antioxidant activity as the samples at 20 mg/mL, were calculated as follows:

ORAC = [C_Trolox_ × (AUC_sample_ − AUC_blank_) × *k*]/(AUC_Trolox_ − AUC_blank_)

where C_Trolox_ is the Trolox concentration, *k* is the sample dilution factor, and AUC is the area under the fluorescence decay curve.

### 2.7. HPLC-DAD Analysis of Carotenoids

The carotenoid extracts were analyzed using HPLC-DAD using a Thermo Spectra Series pump with a UV 6000 LP DAD (Thermo Scientific, Waltham, MA, USA). The chromatographic separation was performed on a reverse-phase C-30 Develosil column (250 × 4.6 mm i.d., 5 μm, Nomura Co., Kyoto, Japan) using the following mobile phase solvents: A (MeOH: H_2_O, 97:3 *v/v*); B (MTBE). The optimized gradient program was reported in detail in a previous paper [12]. Excalibur software (Chromatographic Specialties Inc., Brockville, ON, Canada) was used for data acquisition. The quantification of carotenoids was carried out using calibration curves of standard solutions of β-carotene, lutein (0.24–5.90 μg/mL), and zeaxanthin dipalmitate (0.95–95.0 μg/mL). The validation of the method was reported in a previous paper [12]. Lutein was chosen for the quantification of non-esterified carotenoids (expressed as μg Lutein Equivalents/g, μg LE/g), being the most representative compound of this type of carotenoids, while zeaxanthin dipalmitate was chosen for the quantification of esterified carotenoids (expressed as μg Zeaxanthin Dipalmitate Equivalents/g, μg ZDE/g).

### 2.8. LC-HRMS Analysis for Carotenoid Structural Confirmation

For confirming the carotenoids present in the pumpkin extracts, a UHPLC system Agilent Technologies mod. 1260 Infinity composed of a degasser, a binary pump, an au-tosampler, a thermostated column compartment, and a DAD coupled to a quadrupole-time of flight (Q-TOF) mass spectrometer Agilent mod. 6530 Accurate-Mass Q-TOF LC/MS with a Dual Jet Stream ESI (Dual AJS ESI) (Agilent Technologies, Santa Clara, CA. USA) was employed. The chromatographic separation was carried out on a Zorbax Eclipse Plus C18 (100 × 2.1 mm i.d., 1.8 μm, Agilent Technologies, Santa Clara, CA, USA) maintained at 30 °C.

The mobile phase solvents were: A (H_2_O:FA, 99.9:0.1 *v/v*); B (IPA:ACN:MeOH, 60:20:20 *v/v* with 0.1% FA). The gradient program was: 0 min 45% A and 55% B maintained for 1 min, 3 min 20% A and 80% B, 8 min 10% A and 90% B, 11 min 2% A and 98% B, 16 min 100% B maintained for 19 min. The elution was performed at a flow rate of 0.27 mL/min. For carotenoid analysis, the DAD was set at 447 nm, while spectra were acquired in the range 190–640 nm. The LC-MS analysis was conducted in positive ion mode (*m/z* range 50–1700 amu) at a scan speed of 1.5 spectrum/s. For accurate mass measurements, two reference masses (using a calibrating solution infused continuously through the second nebulizer) having *m/z* 121.050873 and 922.009798 were used.

The following conditions of the Dual AJS ESI source were used: capillary voltage 4000 V, fragmentor voltage 110 V, gas temperature 325 °C, drying gas flow 12 L/min, nebulizer pressure 35 psi, skimmer 60 V, octapole RF 750 V, sheath gas temperature 375 °C, sheath gas flow 12 L/min, and nozzle 0 V. Data were acquired in all-ion mode by acquiring the chromatogram in full scan collision energies of 0 V, 20 V, and 40 V. The analyses and data processing were performed using the Agilent MassHunter software version 10.1 (Santa Clara, CA, USA). The identification of the carotenoids was conducted by comparing experimental spectra with those present in online libraries of MS and MS/MS spectra (Human Metabolome Database or HMDB and MoNA MassBank of North America) and what was reported in previous papers [18,19].

### 2.9. Statistical Analysis

All of the analytical determinations (color, yield, TCC, antioxidant, and chromatographic data) were performed in triplicate, and the results, expressed as mean value ± standard deviation (SD), were reported on dry weight. Microsoft Excel 2016 (Microsoft Corporation, Redmond, WA, USA) was used for data analysis. Statistical significance among the eight varieties was measured using one-way analysis of variance (ANOVA) followed by Tukey’s honestly significant difference post hoc. Values with *p* < 0.01 were considered significant. The differences between the two pumpkin species, *C. maxima* and *C. moschata*, were measured using Student’s *t*-test. A *p*-value < 0.05 was considered to be significant. OriginPro 9.0 (OriginLab Corporation, Northampton, MA, USA) was used as statistical software.

## 3. Results and Discussion

### 3.1. Color Analysis of Pumpkin Peels and Filaments

Table 1a shows the results of the CIELAB and CIELCH color parameters of peels of pumpkins from *C. moschata* (Butternut, Lunga di Napoli, Moscata di Provenza, and Violina rugosa) and *C. maxima* (Delica, Delica vanity, Hokkaido, and Mantovana) species. The results of the color analysis of pumpkin filaments are reported in Table 1b.

The CIELAB scale, used to express colorimetric results, covers the entire range of human color perception, and it is based on the opponent color model of human vision. It expresses color as three values: L* for lightness, a* for red–green opponent colors, and b* for yellow–blue opponent colors. Regarding the CIELCH parameters, the C* value represents color saturation, while hue angle (H* value) reflects the chromaticity or tone of color.

On the basis of the color measurement carried out on these two types of byproducts, a wide variation in the range of the obtained results can be observed.

As regards the L* (lightness) parameter, mean values of 52.67 and 32.75 were found for the peel and filaments of *C. moschata* species, while 46.00 and 33.75 were found for *C. maxima*, respectively. As regards the b* (yellowness) parameter, mean values of 38.67 and 29.58 were found for the peel and filaments of *C. moschata* species, while 42.33 and 29.42 were found for *C. maxima*, respectively. An opposite trend (higher values for filaments in respect to peel) can be observed for the a* (redness) parameter, with the exception of Hokkaido with an orange–red peel that showed the highest value, while the lowest values were found for peel from Butternut, Delica, and Delica vanity varieties. The ranges of C* values were 35.03–46.00 for peel and 22.85–43.19 for filaments. The value of H* was in the first quadrant of hue angle (0–90) and located in the range of red hue to yellow hue.

Statistically different results (*p* ≤ 0.01) were obtained from the comparison between L* values of the peel and filaments of a same species (*C. moschata*). The same result was obtained for the a*, b*, C*, and H* parameters. *C. maxima* species did not show significant differences between the peel and filaments (L*, a*, b*, C*), with the exception of the H* parameter (*p* < 0.05).

A wide variation in the colorimetric data has been reported by other authors. Norfezah et al. (2011) studied pumpkin waste (peel and seeds) to add to extruded snack foods, and found values of 53.42, −0.55, and 24.91 for L*, a*, and b*, respectively, for the peel of Crown pumpkin (*C. maxima*) [20]. They found higher values for the seeds of the same pumpkin variety (62.14, 3.42, and 19.16 for L*, a*, and b*, respectively). Very different values were reported by Sharma and Bhat (2021) for the peel extract of two *C. maxima* varieties: 4.52, 2.92, and 3.17 for Gold Nugget, and 1.87, 5.48, and 3.01 for Amoro F1 [11].

### 3.2. TCC and Antioxidant Activities of Peel and Filament Extracts

Table 2a,b shows the moisture (%) of powders, the yield (%) of extraction, TCC, and in vitro antioxidant activity (ABTS and ORAC assays) of peel and filament extracts, respectively. The moisture % value showed values between 85.64% and 92.60% for *C. moschata* species, and between 75.20 and 85.27% for *C. maxima* species in the peel, while higher moisture values were found in the filaments, even if the differences were not significant (*p* > 0.05).

As regards the % yield, it is possible to observe that the values of peels (mean value: 3.06% for *C. moschata*, 4.57% for *C. maxima*) were higher than those for filaments (mean value: 2.16 and 2.01%, respectively) considering all pumpkin species (*p* > 0.05). The yield % value ranged from 1.69% of Delica vanity to 5.67% of Hokkaido for peels, and from 1.53% to 2.68% for filaments.

Different spectrophotometric characterizations were carried out on carotenoid extracts to determine both the TCC and antioxidant properties using ABTS and ORAC assays. The TCC results of peel extracts changed over the range 148.30 μg/g of Butternut (*C. moschata*) to 1321.53 μg/g of Hokkaido (*C. maxima*), while the values were generally higher (*p* ≥ 0.05) for the filaments (from 234.15 μg/g of Lunga di Napoli to 1655.62 μg/g of Butternut). Considering the same pumpkin variety, only Butternut, Violina rugosa, and Delica vanity showed values of TCC of filaments higher than those of peels.

The literature around spectrophotometric and chromatographic data of carotenoids from pumpkin waste is very lacking, especially when it concerns filaments, and generally focuses on the phenol fraction or other bioactive compounds [5,21,22]. In addition, it must be highlighted that the composition of pumpkin waste can change based on pumpkin variety, harvesting time, conditions, and methods of extraction [23,24,25]. Hussain et al. [4] reported a TCC value of 23.7 mg/100 g powder. The TCC values of extracts from two varieties of *C. maxima* ranged from 16.21 μg/g of oil extracts of a conventional extraction method to 34.94 μg/g of oil extract of MAE, up to 38.03 μg/g of oil extract for UAE using corn oil [11]. Salami et al. [5], instead, found values of 11.48 and 15.22 mg carotene/100 g extract (pumpkin peel extract) obtained using supercritical fluid extraction or subcritical water extraction, respectively.

As regards the total antioxidant capacity, two complementary spectrophotometric methods were carried out. The ABTS assay is useful for determining the extract’s ability to reduce ABTS^•+^ by electron donation, and the ABTS values of peel extracts ranged from 406.81 μg TE/g of Violina rugosa to 2133.96 μg TE/g of Delica, showing the lowest value for *C. moschata* species, and the highest values for *C. maxima* species. The ORAC assay, instead, measures the fluorescence of the molecular probe, and the ORAC values of peel extracts ranged from 2380.94 to 8196.97 μg TE/g for the same pumpkin varieties (Violina rugosa and Delica, respectively), in line with the ABTS results. As regards the filaments, Violina rugosa and Delica vanity showed the highest values of ABTS and ORAC, while Mantovana showed the lowest values. In line with the TCC trend, the ABTS and ORAC values of filaments were also higher for Butternut, Violina rugosa, and Delica vanity pumpkin varieties.

Statistically different results (*p* < 0.05) were obtained from the comparison between ABTS values of filaments of *C. moschata* and *C. maxima* species.

For a valid comparison of antioxidant properties of peel and filament extracts with the literature data, the same shortcomings highlighted for TCC must be taken into consideration. In addition, different units of measurement are used to express the results of antioxidant assays, so there are objective difficulties in making comparisons between the results of different investigations. For example, Sharma and Bhat [11] reported values of DPPH (1,1-diphenyl-2-picrylhydrazyl) % inhibition from 55.95% to 93.53%, using a conventional extraction method or the UAE method, for two pumpkin varieties belonging to *C. maxima* species. Bahramsoltani et al. [6] reported a DPPH IC50 value of 4.015 mg/mL for a hydroalcoholic extract of *C. moschata* Duchesne peel. Values ranging from 2.470 to 4.524 μg TE/g DW for ABTS and from 0.947 to 3.333 μg TE/g DW for DPPH were reported for *Cucurbita* spp. peel extracts [20].

### 3.3. Carotenoid Composition of Peel and Filament Extracts

**Figure 1:**
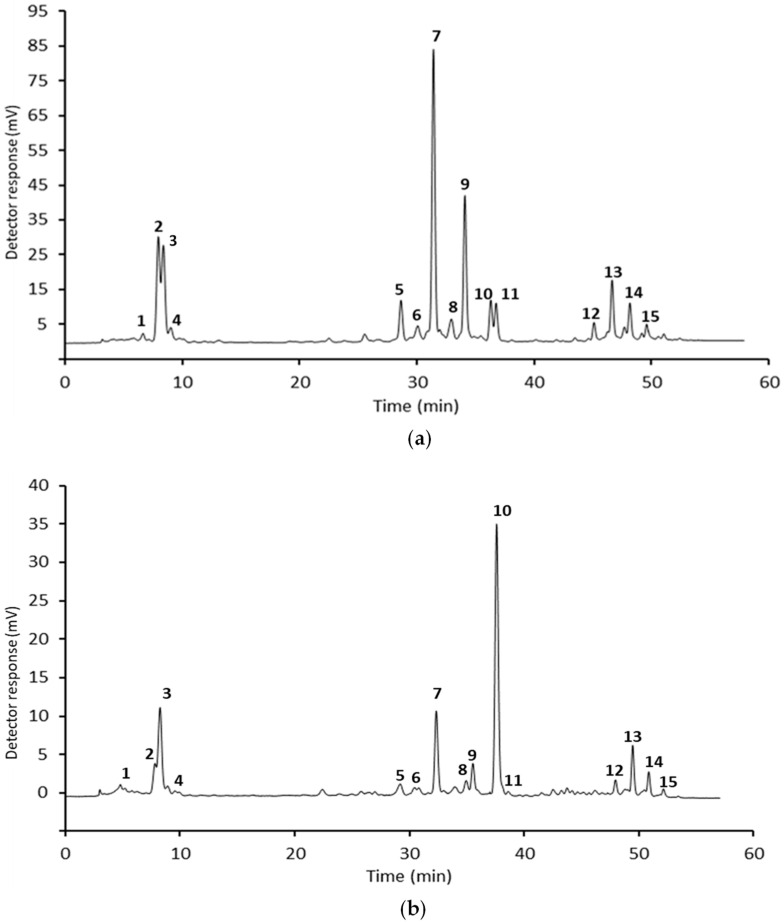
(**a**). A typical HPLC-DAD chromatographic profile of Hokkaido peel. (**b**). A typical HPLC-DAD chromatographic profile of Hokkaido filaments. 1, violaxanthin; 2, antheraxanthin; 3, lutein; 4, zeaxanthin; 5, violaxanthin myristate; 6, lutein palmitate; 7, antheraxanthin myristate; 8, antheraxanthin palmitate; 9, β-carotene; 10 violaxanthin dimyristate; 11, antheraxanthin dilaurate; 12, lutein laurate myristate; 13, lutein dimyristate; 14, lutein myristate palmitate; 15, lutein dipalmitate.

The quantification of carotenoids of the investigated extracts was carried out using HPLC-DAD analysis based on calibration curves built up using standard solutions of β-carotene, lutein, and zeaxanthin dipalmitate [12]. As an example, Figure 1a,b shows, respectively, the chromatographic profile of the carotenoids of Hokkaido peel and filaments, with the peaks confirmed using HPLC-QTOF-MS.

Table 3a,b shows the carotenoid quantification of peel and filament extracts, respectively. The non-esterified carotenoids were represented by violaxanthin, antheraxanthin, lutein, and zeaxanthin, while the esterified carotenoids were constituted of their mono- and di- esters (palmitate, myristate, laurate).

The content of β-carotene varied in a wide range both for peel (6.91–119.94 μg/g) and filaments (30.10–640.99 μg/g). It can be observed that, generally, peel showed a lesser β-carotene content in respect to the filaments of the same variety. The results showed that Delica was the variety with the highest content of β-carotene in the peel, while the respective filaments showed the lowest values. Among the first works concerning the identification and quantification of carotenoids in pumpkin peel, the research of Kreck et al. [26] must be mentioned. They found that β-carotene was the main carotenoid in eight pumpkin cultivars (Muskat, Bischofsmütze, Baby Bear, Butternut, Rouge, Neon, and Hokkaido) belonging to *C. maxima* species. As regards the β-carotene content of peel, Song et al. [27] reported a value of 18.92 μg/g for *C. moschata*, while Hussain et al. [4] found 46 μg/g for *C. maxima*. Both the peel and filaments obtained from the Moscata di Provenza cultivar were characterized by a high and similar content of β-carotene as well as α-carotene. A similar trend was also reported by Kulczyński and Gramza-Michałowska for the pulp of other cultivars belonging to *C. moschata* species [23]. Statistically different results (*p* < 0.05) were obtained from the comparison between the β-carotene contents of the peel and filaments of *C. moschata* species.

It is important to remember that some carotenoids such as ß-carotene are metabolic precursors of vitamin A (pro-vitamin A activity), which is important for the human and mammalian visual system and for cell growth. Moreover, the daily dietary consumption of fruits and vegetables rich in carotenoids has been linked to numerous health benefits, including a reduction in the risk of cardiovascular disease, eye disorders, and several types of cancer (prostate, breast, colorectal) [28].

In addition to the cyclic carotene, i.e., β-carotene, dihydroxycarotenoids such as lutein and zeaxanthin and dihydroepoxycarotenoids such as violaxanthin and antheraxanthin were also found. As regards *C. moschata* species, the content of non-esterified carotenoids expressed as μg LE/g ranged from 10.41 to 22.62 for peel, and from 9.58 to 28.33 for filaments. In some cases, the highest values were found in *C. maxima* species as, for example, 376.66 μg LE/g for Hokkaido peel, and 240.71 μg LE/g for the filaments of Delica vanity. Generally, it can be affirmed that lutein is the main carotenoid after β-carotene, as can be seen for the peel and filaments of pumpkins belonging to the *C. moschata* species which seem to contain only lutein. On the contrary, the waste of *C. maxima* species showed violaxanthin and antheraxanthin, in addition to lutein. It must be underlined that lutein plays an importan nutritional role in a daily varied diet, confering promising benefits against numerous health issues, including neurological disorders, eye diseases, skin irritation, etc. [29]. In a recent paper, Song et al. [27] applied the UAE method to pumpkin (*C. moschata* Duch. cv. Miben) peel, in order to avoid the occurrence of high isomerization of total *trans* carotenoids, and included *trans* lutein vs. *cis* isomers during conventional extraction.

Interestingly, the analyzed waste contained a high quantity of esterified carotenoids, including violaxanthin, anteraxanthin, and lutein laurate, myristate, and palmitate, as mono-, but also as di-, esterified compounds. This result confirms what is reported in the literature, namely that in fruits and vegetables such as persimmon, pepper, and squash, xanthophylls are mostly found in esterified form [30]. In this work, the highest content of esterified carotenoids (13,272.29 μg ZDE/g) was found in Hokkaido peel, while the lowest was found in Butternut. Also, Hokkaido filaments are a good source of esterified carotenoids (2292.00 μg ZDE/g), even if the highest content was found in Butternut (14,255.80 μg ZDE/g). It can, therefore, be highlighted that if the peel of a particular pumpkin variety is a good source of esterified carotenoids, the same thing does not occur for the respective filaments.

The esterification of carotenoids has been correlated with greater stability and accumulation, and potentially increased bioavailability; in fact, it has been reported that the in vivo absorption of carotenoids is better if they are esterified rather than non-esterified [31].

### 3.4. Comparative Study between Parameters of the Different Parts of Pumpkins

The present study indicated that the variety/species of pumpkin had a significant influence on the TCC, antioxidant activity, color parameters, and carotenoid composition.

The results of spectrophotometric assays (TCC, ABTS, and ORAC) were processed to evaluate the degree of correlation with chromatographic data for peel (Table 4a) and filaments (Table 4b), respectively.

As regards the peel, good coefficients of determination (R^2^ ≥ 0.6037) were obtained among the TCC, ABTS, ORAC, and not-esterified carotenoids vs. β-carotene evaluated using HPLC-DAD analysis (Table 4a). Furthermore, good correlations (R^2^ ≥ 0.7174) were also found between ABTS and ORAC vs. non-esterified carotenoids or esterified carotenoids. No correlations were found for filaments (Table 4b), with the exception of esterified carotenoids vs. TCC and β-carotene.

Spectrophotometric and chromatographic data were also correlated with colorimetric data. In Table 4a,b, the most interesting correlation values were reported, that is, the L* parameter for peel and a* parameter for filaments.

In addition, a correlation study between peel vs. filaments of the same species was also carried out. The results of spectrophotometric assays (TCC, ABTS, and ORAC) were processed to evaluate the degree of correlation between peel and filaments, and some excellent correlation values were obtained (Table 5).

The best R^2^ values were found by making a comparison between the parameters of a same part of the pumpkin (i.e., peel) for TCC vs. ABTS (R^2^ = 0.8619), TCC vs. ORAC (R^2^ = 0.9103), and ORAC vs. ABTS (R^2^ = 0.9387). The comparison between the same parameter (TCC vs. TCC; ABTS vs. ABTS; ORAC vs. ORAC) of the two different parts of a pumpkin did not show good correlations (R^2^ = 0.1473–0.3969).

## 4. Conclusions

The growing interest of consumers in functional foods and healthy products favors a constant and cutting-edge research, searching for new bioactive compounds to isolate from different vegetable matrices including agri-food waste. Around the world, increasing environmental responsibility has stimulated the research of innovative extraction techniques which aim to develop safer, more sustainable, and affordable procedures. In this work, UAE proved to be an efficient technique for isolating carotenoids from the peel and filaments of pumpkins. The results show that both pumpkin products have good potential to scavenge free radicals, having interesting values of TCC and antioxidant properties. The particularly high content of β-carotene and esterified carotenoids in some pumpkin varieties, as, for example, Butternut and Violina rugosa among *C. moschata* and Delica and Hokkaido among *C. maxima* species, gives this crop interesting potential in the development of new products, such as nutraceuticals and functional foods, by taking advantage of the reuse of the pumpkin peel as well as the filaments.

## Figures and Tables

**Table 1:** (**a**) Color characteristics of pumpkin peel powders; (**b**) Color characteristics of pumpkin filament powders.

**a**					
**Cultivar**	L*	a*	b*	C*	H*
Butternut	55.33 ± 1.15 ^a^	−1.33 ± 0.58 ^a^	35.00 ± 1.00 ^a^	35.03 ± 1.00 ^a^	92.18 ± 0.94 ^a,d^
Lunga di Napoli	51.33 ± 0.58 ^a,c^	-	39.00 ± 0.00 ^b^	39.00 ± 0.00 ^b^	90.00 ± 0.00 ^a,b^
Moscata di Provenza	52.00 ± 0.00 ^a,c^	1.33 ± 0.58 ^b^	38.00 ± 0.00 ^b^	38.03 ± 0.02 ^b^	87.99 ± 0.87 ^b^
Violina rugosa	52.00 ± 2.65 ^a,c^	3.33 ± 0.58 ^c^	42.67 ± 1.15 ^c,d^	42.80 ± 1.13 ^c^	85.52 ± 0.84 ^c^
Delica	39.33 ± 0.58 ^b^	−0.33 ± 0.58 ^a,d^	40.00 ± 0.00 ^b,c^	40.00 ± 0.01 ^b^	90.48 ± 0.83 ^a^
Delica vanity	50.00 ± 1.73 ^c,e^	−2.67 ± 0.58 ^a^	39.00 ± 1.00 ^b^	39.09 ± 1.03 ^b^	93.90 ± 0.77 ^d^
Hokkaido	45.33 ± 0.58 ^d,e^	10.33 ± 0.58 ^d^	44.33 ± 0.58 ^d^	45.52 ± 0.69 ^c,d^	76.89 ± 0.54 ^e^
Mantovana	49.33 ± 1.53 ^c,e^	-	46.00 ± 1.00 ^d^	46.00 ± 1.00 ^d^	90.00 ± 0.00 ^a,b^
**b**					
**Cultivar**	L*	a*	b*	C*	H*
Butternut	31.00 ± 0.00 ^a^	12.33 ± 0.58 ^a^	29.33 ± 0.58 ^a,c^	31.83 ± 0.46 ^a,c^	67.19 ± 1.21 ^a^
Lunga di Napoli	35.33 ± 1.15 ^b^	10.00 ± 0.00 ^b^	31.00 ± 1.00 ^a,c^	32.63 ± 0.96 ^a^	72.11 ± 0.54 ^b^
Moscata di Provenza	32.33 ± 0.58 ^a^	9.00 ± 0.00 ^b^	26.67 ± 0.58 ^b^	28.14 ± 0.55 ^b^	71.35 ± 0.38 ^b,c^
Violina rugosa	32.33 ± 0.58 ^a^	12.67 ± 0.58 ^a^	31.33 ± 0.58 ^a^	33.80 ± 0.67 ^a^	67.99 ± 0.80 ^a,c^
Delica	30.00 ± 1.00 ^a^	8.33 ± 0.58 ^b,c^	26.67 ± 1.15 ^b,c^	27.94 ± 1.27 ^b^	72.66 ± 0.41 ^b^
Delica vanity	29.33 ± 1.15 ^a^	10.00 ± 1.00 ^b^	27.33 ± 0.58 ^b^	29.12 ± 0.64 ^b,c^	69.92 ± 1.90 ^a,b,c^
Hokkaido	24.00 ± 1.00 ^c^	9.00 ± 1.00 ^b^	21.00 ± 1.73 ^d^	22.85 ± 1.94 ^d^	66.82 ± 1.25 ^a^
Mantovana	51.67 ± 0.58 ^d^	6.67 ± 0.58 ^c^	42.67 ± 0.58 ^e^	43.19 ± 0.53 ^e^	81.12 ± 0.82 ^d^

L*, color lightness; a*, degree of greenness (negative)–redness (positive); b*, degree of blueness (negative)–yellowness (positive); C*, chroma; H*, hue angle. Different letters in each column indicate significant differences with *p*-value < 0.01. Above are the data for *C. moschata*, below are those for *C. maxima* species.

**Table 2:** (**a**) Moisture (%) of pumpkin peel, yield (%) of extraction, TCC, and in vitro antioxidant activity (ABTS and ORAC assays) of pumpkin peel extracts; (**b**) moisture (%) of pumpkin filaments, yield (%) of extraction, TCC, and in vitro antioxidant activity (ABTS and ORAC assays) of pumpkin filament extracts.

**a**					
**Cultivar**	**Moisture (%)**	**Yield ** **(%)**	**TCC** **(µg β-CE/g)**	**ABTS** **(μg TE/g)**	**ORAC** **(μg TE/g)**
Butternut	87.94 ± 4.23 ^a,b^	2.87 ± 0.00 ^a^	148.30 ± 5.96 ^a^	557.93 ± 3.43 ^a^	3568.60 ± 87.39 ^a,b^
Lunga di Napoli	92.60 ± 5.89 ^a^	4.34 ± 0.00 ^b^	336.06 ± 22.08 ^b^	1041.40 ± 14.96 ^b^	4203.88 ± 108.53 ^a^
Moscata di Provenza	91.90 ± 5.21 ^a^	2.66 ± 0.00 ^a^	269.18 ± 4.22 ^a,b^	950.37 ± 44.79 ^b^	3068.01 ± 106.00 ^b,c^
Violina rugosa	85.64 ± 4.76 ^a,b,c^	2.38 ± 0.00 ^a^	305.97 ± 25.17 ^b,d^	406.81 ± 3.69 ^c^	2380.94 ± 90.14 ^c^
Delica	78.03 ± 3.89 ^b,c^	5.65 ± 0.00 ^c^	1089.95 ± 93.98 ^c^	2133.96 ± 7.53 ^d^	8429.65 ± 105.58 ^d^
Delica vanity	75.20 ± 4.01 ^c^	1.69 ± 0.00 ^d^	184.03 ± 12.52 ^a,d^	602.01 ± 49.16 ^a^	2450.35 ± 54.50 ^c^
Hokkaido	85.27 ± 5.14 ^a^	5.67 ± 0.00 ^c^	1321.53 ± 125.36 ^e^	1906.21 ± 53.15 ^e^	8196.97 ± 820.22 ^d^
Mantovana	81.01 ± 5.02 ^a,b,c^	5.28 ± 0.00 ^c^	510.88 ± 12.97 ^f^	1049.47 ± 64.36 ^b^	4425.83 ± 100.78 ^a^
**b**					
**Cultivar**	**Moisture (%)**	**Yield ** **(%)**	**TCC ** **(µg β-CE/g)**	**ABTS** **(μg TE/g)**	**ORAC** **(μg TE/g)**
Butternut	95.51 ± 4.87 ^a,b^	2.16 ± 0.00 ^a^	1655.62 ± 67.32 ^a^	1171.01 ± 63.92 ^a^	6637.26 ± 90.92 ^a^
Lunga di Napoli	96.72 ± 6.03 ^a,b^	1.88 ± 0.00 ^a,d^	234.15 ± 14.40 ^b^	880.42 ± 66.24 ^b^	3918.79 ± 40.35 ^b^
Moscata di Provenza	96.71 ± 5.98 ^a^	1.98 ± 0.00 ^a^	926.48 ± 64.83 ^c^	850.25 ± 29.19 ^b,d^	5671.89 ± 60.42 ^c^
Violina rugosa	90.73 ± 4.75 ^b,c^	2.62 ± 0.00 ^b^	1644.08 ± 20.35 ^a^	1452.66 ± 30.65 ^c^	8767.71 ± 100.34 ^d^
Delica	88.18 ± 3.52 ^c^	2.17 ± 0.00 ^a^	481.99 ± 29.20 ^d^	748.83 ± 40.50 ^d^	4871.40 ± 219.25 ^e^
Delica vanity	89.85 ± 4.52 ^c^	2.68 ± 0.00 ^b^	1006.00 ± 78.72 ^c^	1490.77 ± 69.74 ^c^	7798.34 ± 244.90 ^f^
Hokkaido	86.86 ± 4.08 ^c^	1.53 ± 0.00 ^c^	504.47 ± 26.66 ^d^	878.55 ± 4.72 ^b^	5969.67 ± 79.57 ^c^
Mantovana	84.96 ± 5.62 ^c^	1.66 ± 0.00 ^c,d^	323.25 ± 24.97 ^b^	509.74 ± 33.42 ^e^	2487.11 ± 100.30 ^g^

Data are reported as mean value ± standard deviation (SD) of three independent measurements (*n* = 3) and are expressed on dry weight (DW). β-CE, β-carotene equivalents; TE, Trolox equivalents. Different letters in each column indicate significant differences with *p*-value < 0.01. Above are the data for *C. moschata*, below are those for *C. maxima* species.

**Table 3:** (**a**) Contents of non-esterified carotenoids, β-carotene, and esterified carotenoids of pumpkin peel extracts; (**b**) Contents of non-esterified carotenoids, β-carotene, and esterified carotenoids of pumpkin filament extracts.

**a**			
**Cultivar**	**Non-Esterified Carotenoids** **(μg LE/g)**	**β-Carotene** **(μg/g)**	**Esterified Carotenoids ^^^** **(** **μg ZDE/g)**
Butternut	10.41 ± 0.02 ^a^ *	6.91 ± 1.02 ^a^	885.82 ±5.84 ^a^
Lunga di Napoli	22.62 ± 0.41 ^a^ *	26.25 ± 1.65 ^b^	1433.89 ± 0.93 ^a^
Moscata di Provenza	13.46 ± 1.21 ^a^ *	17.36 ± 1.08 ^c #^	1283.72 ± 101.47 ^a^
Violina rugosa	11.78 ± 0.45 ^a^ *	30.00 ± 1.47 ^b,f^	2254.64 ± 126.05 ^b^
Delica	376.66 ± 24.51 ^b $^	119.94 ± 8.51 ^d^	8237.85 ± 363.37 ^c^
Delica vanity	30.76 ± 0.49 ^a^ *	10.59 ± 1.96 ^a,c^	1533.63 ± 35.00 ^a,e^
Hokkaido	329.53 ± 21.65 ^b $^	50.97 ± 2.35 ^e^	13272.29 ± 878.41 ^d^
Mantovana	109.13 ± 8.82 ^c^ *	37.85 ± 3.11 ^f^	2161.96 ± 183.21 ^b,e^
**b**			
**Cultivar**	**Non-Esterified Carotenoids** **(μg LE/g)**	**β-Carotene** **(μg/g)**	**Esterified Carotenoids ^^^** **(** **μ** **g ZDE/g)**
Butternut	22.73 ± 0.31 ^a^ *	640.99 ± 2.70 ^a^	14,255.80 ± 270.40 ^a^
Lunga di Napoli	9.58 ± 0.53 ^b^ *	54.55 ± 3.83 ^b^	1755.28 ± 122.40 ^b^
Moscata di Provenza	14.59 ± 0.45 ^b^ *	542.61 ± 2.77 ^c #^	3059.58 ± 17.79 ^c^
Violina rugosa	28.33 ± 2.26 ^a^ *	613.70 ± 44.02 ^a^	9635.20 ± 632.2 ^d^
Delica	135.49 ± 1.47 ^c $^	30.11 ± 2.30 ^b^	3132.34 ± 112.32 ^b^
Delica vanity	240.71 ± 7.56 ^d &^	236.38 ± 13.46 ^d^	5712.38 ± 5.44 ^e^
Hokkaido	77.79 ± 3.48 ^e &^	110.25 ± 7.53 ^e^	2292.00 ± 117.88 ^b^
Mantovana	57.31 ± 0.51 ^f &^	30.10 ± 0.79 ^b^	2309.17 ± 5.57 ^b^

The data are reported as mean value ± SD. LE, Lutein Equivalents; ZDE, Zeaxanthin Dipalmitate Equivalents; * only lutein; ^$^ sum of violaxanthin, antheraxanthin, and lutein; ^&^ antheraxanthin and lutein; ^^^ violaxanthin myristate, lutein palmitate, antheraxanthin myristate, antheraxanthin palmitate, violaxanthin dimyristate, antheraxanthin dilaurate, lutein laurate myristate, lutein dimyristate, lutein myristate palmitate, lutein dipalmitate; ^#^ β-carotene + α-carotene. Different letters in each column indicate significant differences with *p*-value < 0.01. Above are the data for *C. moschata*, below are those for *C. maxima* species.

**Table 4:** (**a**) Correlation analysis (coefficient of determination, R^2^) of pumpkin peel extracts; (**b**) Correlation analysis (coefficient of determination, R^2^) of pumpkin filament extracts.

**a**				
**Peel**	**Non-Esterified Carotenoids**	**β-Carotene**	**Esterified ** **Carotenoids**	L*
TCC	0.2461	0.6037	0.1451	0.7239
ABTS	0.8975	0.7075	0.7174	0.8065
ORAC	0.9350	0.6824	0.7951	0.7272
Non-esterified carotenoids	-	0.7663	0.8249	0.8646
β-carotene	0.7663	-	0.414	0.8781
Esterified carotenoids	0.8249	0.4141	-	0.5693
**b**				
**Filaments**	**Non-Esterified Carotenoids**	**β-Carotene**	**Esterified ** **Carotenoids**	a*
TCC	0.0102	0.3057	0.8913	0.7082
ABTS	0.1021	0.4991	0.3308	0.6648
ORAC	0.0622	0.8582	0.3842	0.6182
Non-esterified carotenoids	-	0.0787	0.0578	0.0444
β-carotene	0.0787	-	0.9045	0.7696
Esterified carotenoids	0.0578	0.9045	-	0.5924

**Table 5:** Correlation analysis (coefficient of determination, R^2^) between byproduct (peel vs. filament) extracts.

Peel		TCC	ABTS	ORAC
TCC	Peel	-	0.8619	0.9103
	Filaments	0.2737	0.2229	0.0653
ABTS	Peel	0.8619	-	0.9387
	Filaments	0.4555	0.3969	0.2038
ORAC	Peel	0.9103	0.9387	-
	Filaments	0.3177	0.3167	0.1473

## Data Availability

The data used to support the findings of this study can be made available by the corresponding author upon request.
